# The nuclear entry of the aryl hydrocarbon receptor (AHR) relies on the first nuclear localization signal and can be negatively regulated through IMPα/β specific inhibitors

**DOI:** 10.1038/s41598-023-47066-z

**Published:** 2023-11-11

**Authors:** Rashad Haidar, Reneh Shabo, Marie Moeser, Andreas Luch, Josephine Kugler

**Affiliations:** 1 Department of Chemical and Product Safety, German Federal Institute for Risk Assessment (BfR), Berlin, Germany; 2 Department of Biology, Chemistry and Pharmacy, Institute of Pharmacy, Freie Universität Berlin, Berlin, Germany

**Keywords:** Biochemistry, Biotechnology, Cell biology, Molecular biology

## Abstract

The human aryl hydrocarbon receptor (AHR) undergoes continuous shuttling between nucleus and cytoplasm. Binding to exogenous or endogenous ligands promotes its rapid nuclear import. The proposed mechanism for the ligand-dependent import is based on exposing the bipartite nuclear localisation signal (NLS) to members of the importin (IMP) superfamily. Among this, the molecular interactions involved in the basal import still need to be clarified. Utilizing fluorescently fused AHR variants, we recapitulated and characterized AHR localization and nucleo-cytoplasmic shuttling in living cells. Analysis of AHR variants carrying NLS point mutations demonstrated a mandatory role of first (_13_RKRRK_17_) and second (_37_KR-R_40_) NLS segments on the basal import of AHR. Further experiments indicated that ligand-induced import is mainly regulated through the first NLS, while the second NLS is supportive but not essential. Additionally, applying IMPα/β specific inhibitors, ivermectin (IVM) and importazole (IPZ), slowed down the ligand-induced import and, correspondingly, decreased the basal nuclear accumulation of the receptor. In conclusion, our data show that ligand-induced and basal nuclear entry of AHR rely on the same mechanism but are controlled uniquely by the two NLS components.

## Introduction

The aryl hydrocarbon receptor (AHR) is a ligand-dependent transcription factor, which was initially discovered due to its contribution in mediating the toxicity of exogenous environmental toxicants, like 2,3,7,8-tetrachlorodibenzo-*p*-dioxin (TCDD)^1^ and benzo[*a*]pyrene (B[*a*]P)^2^. Exposure to AHR ligands, as for example the synthetic flavonoid β-naphthoflavone (BNF)^3^, induces the expression of xenobiotic metabolizing enzymes (XMEs). These include phase I XMEs such as cytochrome P450-dependent monooxygenase 1A1 (*CYP1A1)* and phase II XMEs such as glutathione *S*-transferase α (*GST-α*)^4^, indicating AHR’s role in xenobiotic metabolism^5^.

More current studies discovered essential physiological roles of AHR by demonstrating that many compounds from endogenous origins, including indole, heme, and tryptophan metabolites^6^^,^^7^, are able to bind and activate the receptor*.* As for example, the endogenous indirubin (IND) is generated from dietary tryptophan by the intestinal and urinary microbiome^8^. It is one of the most potent AHR ligands and exhibits anti-inflammatory effects in vivo^9^. Furthermore, current research reveals that AHR has a more complex role in cell biology. In fact, AHR interacts with other signaling pathways involved in proliferation, apoptosis, and cell cycle regulation^10^^,^^11^.

AHR activation is firmly linked with nuclear transition that depends on a sequence of positively charged amino acids, the nuclear localization signal (NLS)^12^. The NLS comprises two segments (bipartite NLS) and is located within the conserved bHLH (basic helix–loop–helix) domain in the N-terminal part of the protein^13^. According to the classical AHR pathway, unliganded AHR is mainly located in the cytoplasm bound by a chaperon complex consisting of two molecules of HSP90^14^ and single molecules of co-chaperone p23^15^ and hepatitis x-associated protein-2 (XAP2)^16^. Most AHR ligands are able to cross the cell membrane through simple diffusion due to their hydrophobicity^17^. Ligand binding to cytosolic AHR triggers a conformational change that exposes the NLS to a nuclear transporter in order to permit nuclear import^18^. On the molecular level, members of the importin (IMP) superfamily IMPβ1 or its adaptor protein IMPα can recognize the revealed NLS^18^^,^^19^. Thereafter, AHR along with the IMPα/β1 heterodimer reaches the nucleus through the nuclear pore complexes (NPCs). On the nuclear side of NPCs, IMPβ1 binds to RanGTP (Ras-related nuclear protein) subsequently leading to a release of the NLS-cargo^20^.

In the nucleus, AHR releases its chaperons^21^ and builds a stable dimer with the AHR nuclear translocator (ARNT)^22^^,^^23^. The AHR/ARNT heterodimer recruits several co-factors^24^, binds its target sequences, called XRE (xenobiotic response element)^25^, and regulates the expression of AHR target genes. Ultimately, AHR is exported from the nucleus to the cytoplasm to undergo proteasomal degradation marking the end of activation^26^. This nuclear export is established through CRM1 (chromosome region maintenance 1 also known as exportin) that binds the nuclear export signal (NES) of the receptor^27^. In a previous report, we showed that the molecular interactions performed by the receptor in the nucleus, ARNT dimerization and DNA binding, do not alter the nuclear retention after ligand binding^28^.

Analyzing AHR translocation in vitro*,* however, discloses many aspects that are still not completely understood. For example, AHR undergoes constitutive shuttling between the nucleus and cytoplasm^29^^,^^30^, which neither leads to any activation^31^, nor demands HSP90 dissociation^32^. Although most of the effort given on AHR trafficking intends to clarify the ligand-induced transition, the basal shuttling requests further investigation. On this stage, the research done in the last decade revealed many nuclear import inhibitors targeting IMPs^33^. The best characterized ones are importazole (IPZ)^34^ and ivermectin (IVM)^35^^,^^36^. The former revealed interferences with the IMPα/β1 mediated-nuclear entry of NF-κB^33^ and the latter showed import suppressing properties on the hypoxia induced factor (HIF)^37^. Both import inhibitors differ markedly in their mechanism. While IVM inhibits the binding between IMPα and the NLS^38^^,^^39^, IPZ interferes with IMPβ1 and RanGTP binding^40^.

In the current study, we aimed to elucidate the mechanism of AHR translocation in the basal and ligand-induced state in living cells. We confirmed faithful recapitulation of endogenous AHR localization for our EYFP-AHR overexpression system, independent of cell cycle, expression level, or fusion with EYFP. Through point mutations over the entire NLS, importance of both parts of the bipartite NLS for AHR localization has been shown. But surprisingly, these effects are not transferable on the ligand-induced import, which can still occur with 2nd NLS mutants, confirming that the 1st NLS of AHR is mandatory for the nuclear import. Application of the nuclear transport inhibitors IVM and IPZ resulted in a clear reduction of nuclear accumulation of ligand-free and ligand-bound AHR. In summary, our data demonstrated that ligand-induced and basal nuclear entry are two different processes that rely on the same molecular mechanism.

## Results

### EYFP-AHR in MCF-7^ΔAHR^ faithfully recapitulates AHR localization

In our study, we aimed to investigate the nucleo-cytoplasmic translocation of unliganded and ligand bound AHR. To avoid any confounding effects through endogenous AHR, AHR-knockout MCF-7 cells (MCF-7^ΔAHR^) have been used^41^. Transient transfection of MCF-7^ΔAHR^ cells with plasmids coding for EYFP-AHR^WT^ or EYFP-AHR^mut^ allowed real-time tracking of AHR’s subcellular localization in living cells as described previously^28^^,^^31^.

**Figure 1:**
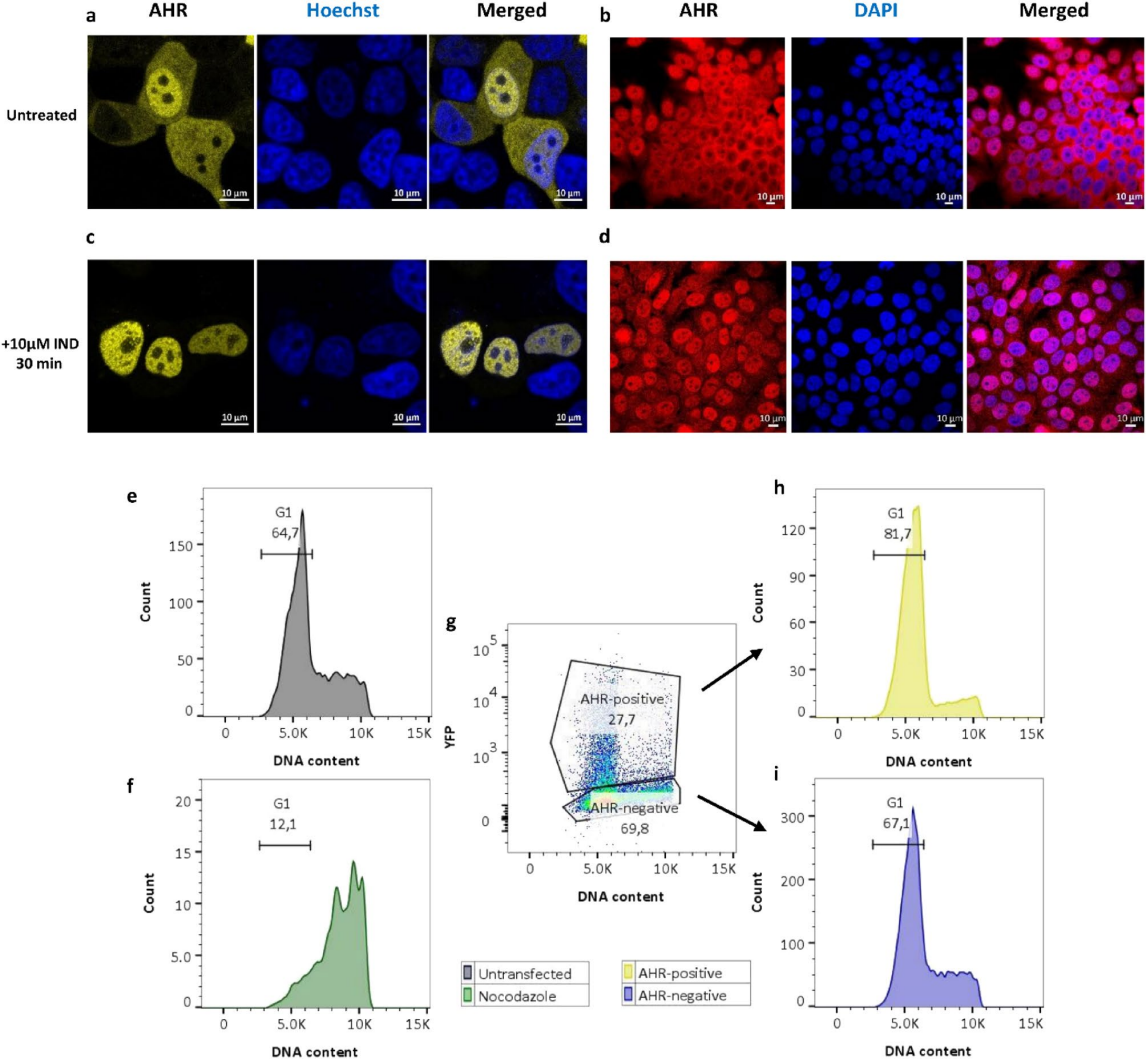
AHR intracellular distribution in MCF-7 cells. Representative images of EYFP-AHR^WT^ transiently transfected in MCF-7^ΔAHR^ cells before (**a**) and after (**c**) 30 min treatment with 10 µM indirubin (IND). Immunofluorescence images of AHR in MCF-7^WT^ cells before (**b**) and after (**d**) 30 min treatment with 10 µM IND. Nuclei are marked either with Hoechst (**a**,**c**) or with DAPI (**b**,**d**). Cell cycle analysis of control cells without (**e**) and with nocodazole (f). EYFP-AHR transfected cells were sorted for expression level (**g**) and subpopulations individually analyzed for cell cycle for EYFP-AHR-positive (**h**) and for EYFP-AHR-negative cells (**i**). Total number of analyzed cells: 7624 (**e**), 1149 (**f**), 18,192 (**g**), 4441 (**h**) and 11,181 (**i**).

**Figure 2:**
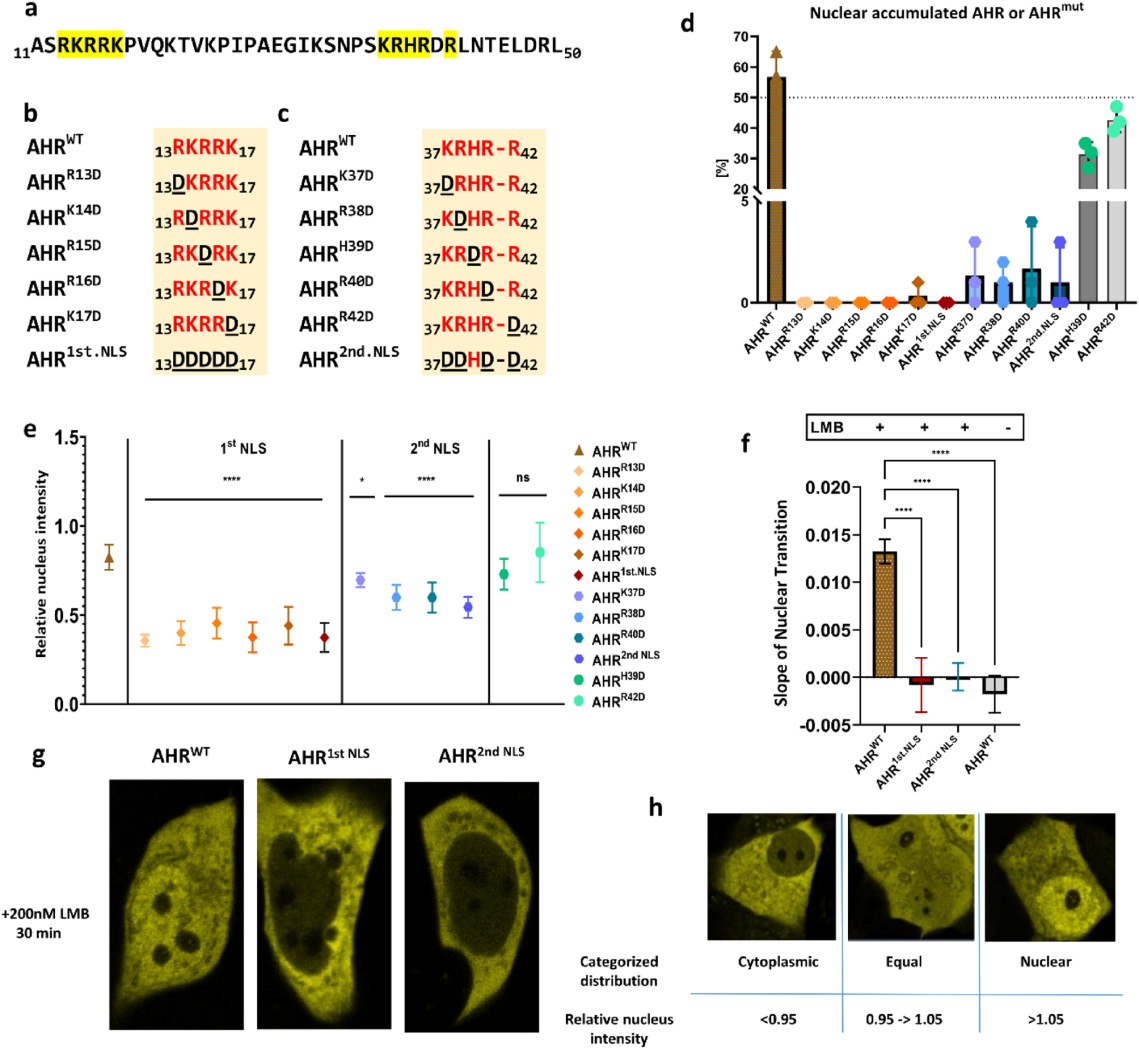
Role of the nuclear localization signal (NLS) on the basal shuttling of AHR. The N-terminal bHLH domain of AHR is indicated as amino acid sequence (**a**). Graphical representation of mutated amino acids in 1st NLS (b) and 2nd NLS (**c**). The percentage of cells with nuclear accumulated AHR^WT^ or AHR^mut^ for 100 randomly chosen cells in the basal state (**d**) according to our classification (**h**). The ratio of the mean fluorescence intensity within the nucleus and the mean fluorescence intensity of the cells in the cytoplasmic state is given as relative nuclear intensity (**e**). Slopes of the nuclear transition of AHR^WT^ or AHR^mut^ after treatment with 200 nM leptomycin B (LMB) for 30 min (**f**). Values represent the mean ± S.D of n = 100 (**d**), n = 10 (**e**), n = 14 for AHR^WT^ and n = 5 for AHR^mut^ or unstimulated AHR^WT^ (**f**). Statistical analysis was performed with a one-way ANOVA, Dunnett's post-test, *p < 0.05, ****p < 0.0001.

Notably, localization of AHR^WT^ in MCF-7^ΔAHR^ cells can be defined in three different states: accumulated in the nucleus, equally distributed or predominantly cytoplasmic (Figs. 1a, 2h and Supplementary Fig. S1). Antibody staining of endogenous AHR in fixed MCF-7^WT^ revealed a similar distribution (Fig. 1b). Treating cells for 30 min with 10 µM IND, however, captured only predominantly nuclear accumulated AHR in both cases (Fig. 1c,d). To assure functional output of EYFP-AHR^WT^ translocation, we verified *CYP1A1* and *CYP1B1* mRNA induction after IND treatment with qPCR (Supplementary Fig. S2).

To exclude an influence of the cell cycle on AHR localization, we determined the cell cycle phase of EYFP-AHR^WT^ transfected MCF-7^ΔAHR^ cells by measuring DNA content. After 48 h of cultivation, roughly 65% of MCF 7^ΔAHR^ cells were in G1 phase (Fig. 1e). With nocodazole as positive control for cell cycle analysis, we shifted the population to mainly G2 phase (Fig. 1f). Interestingly, cells that have been transfected, but were negative for EYFP-AHR expression, exhibited the same distribution as untransfected cells (Fig. 1i). On the other side, the great majority of EYFP-AHR positive cells were in G1 phase (Fig. 1g,h). To further elucidate whether changed cell cycle progression is a result of EYFP-AHR overexpression, we similarly analyzed the cell cycle of cells transiently transfected with EYFP alone. Interestingly, these cells exhibited changed cell cycle distribution as well, leading to the hypothesis, that transfection itself alters cell cycle progression (Supplementary Fig. S3). Overall, transfection of EYFP-AHR^WT^ in MCF-7^ΔAHR^ faithfully recapitulates AHR localization, independent of expression level and cell cycle.

### Impact of individual amino acids of the NLS on the basal import of AHR

Since we sought to investigate the nuclear transition of AHR, we decided to address the impact of NLS components on the basal import of AHR first. Therefore, we mutated every positively charged amino acid of the bipartite NLS individually and generated mutants for the first and the second part of the NLS as well (Fig. 2a–c).

We evaluated the effect of individual amino acids on the intracellular distribution of AHR in MCF-7^ΔAHR^ cells by categorizing at least 100 cells into one of the three described states (Fig. 2h and Supplementary Fig. S4). Consistently, switching the positively charged NLS amino acid arginine to aspartate (AHR^R13D^, AHR^K14D^, AHR^R15D^, AHR^R16D^, AHR^K17D^, AHR^1st.NLS^, AHR^K37D^, AHR^R38D^, AHR^R40D^, and AHR^2nd.NLS^) shifted the AHR compartmentalization to be solely cytoplasmic. On the other hand, similar substitution of the other two positions (AHR^H39D^ and AHR^R42D^) did not alter the typical cellular distribution (Fig. 2d and Supplementary Figs. S5 and S6).

Next, we analyzed the relative nuclear intensity (Fig. 2h) of basal EYFP-AHR^WT^ and EYFP-AHR^mut^, respectively, thus informing about the nuclear retention of AHR in the absence of ligands (Fig. 2e). Our results exhibited a significant decrease in the relative nuclear intensity of NLS mutants compared with primary cytosolic EYFP-AHR^WT^ cells (Fig. 2e and Supplementary Fig. S5). Interestingly, mutations in the first part of the NLS seemed to have a higher impact on the nuclear retention than mutations on the second part of the NLS. Again, EYFP-AHR^H39D^ and EYFP-AHR^R42D^ revealed a comparable nuclear intensity to the wild type (Fig. 2e).

Moreover, we tested whether the basal import can still occur in first and second NLS full mutants after inhibiting nuclear export using leptomycin B (LMB) by measuring the change of relative nuclear intensity over time (Supplementary Fig. S7). Remarkably, the basal import for both mutants was completely repressed after treating the cells with 200 nM LMB for 30 min (Fig. 2f,g and supplementary Fig. S8).

### The ligand-induced nuclear entry of AHR depends primarily on the first part of NLS

Next, we studied the ligand-induced nuclear import of NLS mutants. After treatment with either IND or BNF, we tracked the subcellular location of fluorescent fusion proteins over 15 min.

**Figure 3:**
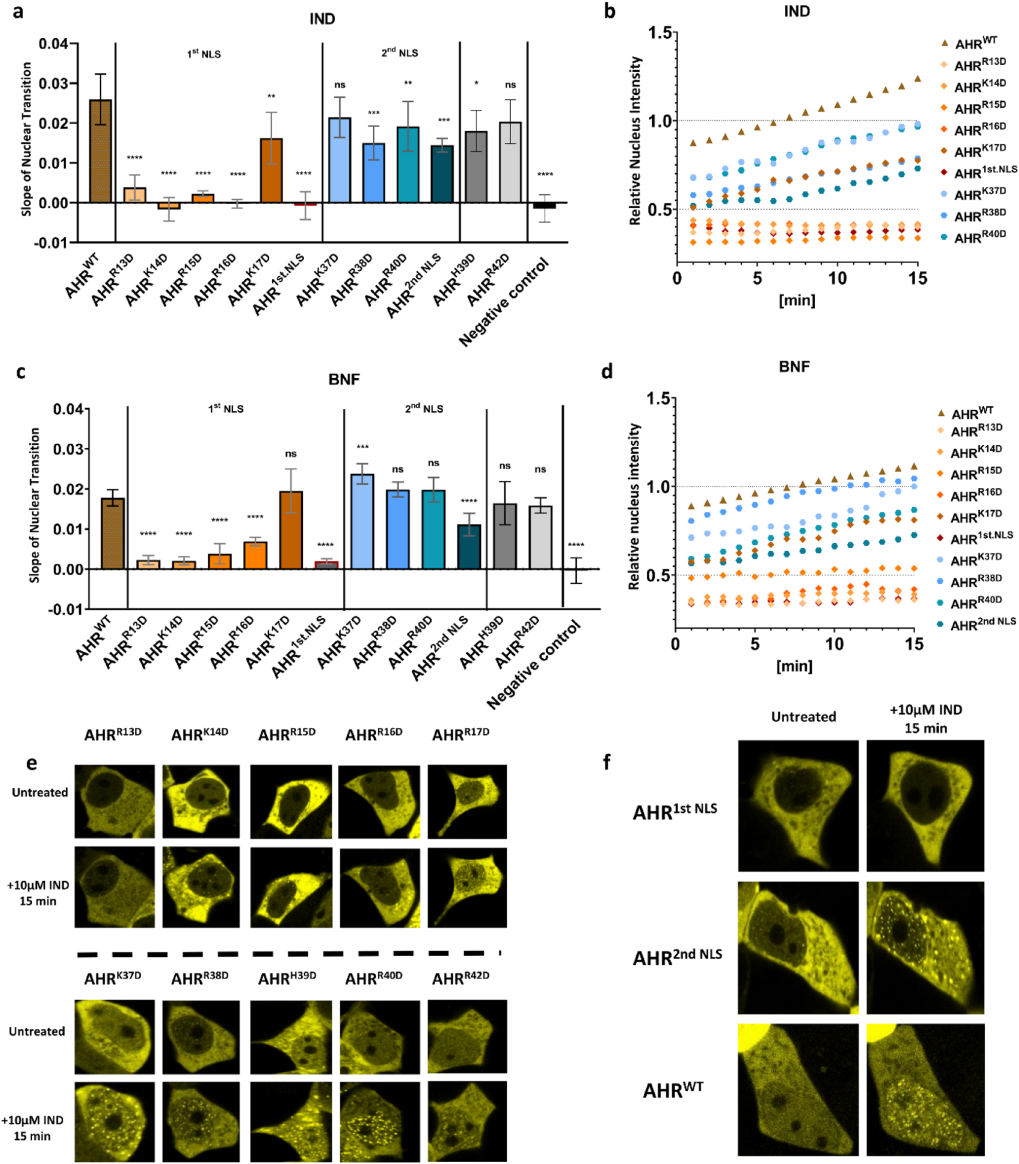
The nuclear entry of AHR depends primarily on the first part of nuclear localization signal (NLS). Slopes of the nuclear transition of EYFP-AHR^WT^ or EYFP-AHR^mut^ after treatment with 10 µM indirubin (IND) (**a**) or 10 µM β-naphthoflavone (BNF) (**c**) for 15 min. Negative control refers to untreated sample transfected with AHR^WT^. Mean of time-lapse measurements after stimulation with 10 μM IND (**b**) or 10 μM BNF (**d**) for 15 min. Representative images of 1st and 2^nd^ NLS point mutants before and after treatment with IND (**e**). Snapshots of AHR^WT^, AHR^1st.NLS^ and AHR^2nd.NLS^ before and after treatment with IND (**f**). Data show the mean ± S.D of n = 12 for AHR^WT^, n = 5 for AHR^mut^ and NC (**a**–**c**). Statistical analysis was performed with a one-way ANOVA, Dunnett's post-test, *p < 0.05, **p < 0.01, ***p < 0.001, ****p < 0.0001.

In response to ligands, all mutants with point mutations in the 1st NLS, except EYFP-AHR^K17D^, revealed a clear reduction of nuclear transition slopes compared to the wild type protein (Fig. 3a–d). In contrast, mutants with point mutations in the 2nd NLS resulted only in a slight reduction of nuclear import after stimulation with IND and showed no effect on the import rate for BNF treatment (Fig. 3a–d). When mutating the entire first or second NLS, respectively, the effect on the nuclear transition slope was similar: mutating the 1st NLS blocked the nuclear transition entirely, whereas a mutation of the 2nd NLS only reduced the rate of nuclear import (Fig. 3a–f). EYFP-AHR^H39D^ and EYFP-AHR^R42D^ had no to very limited effect on the nuclear transition for IND and BNF, respectively, supporting the results from the basal shuttling that these amino acids do not contribute to NLS function (Fig. 3a–e). Interestingly, independent of import rate, nuclear accumulation can take place of ligand activated EYFP-AHR^WT^ or EYFP-AHR^mut^, leading to the formation of protein clusters within the nucleus (Fig. 3e, f and supplementary Fig. S3).

### IPZ and IVM reduce nuclear accumulation of AHR

Taken together, the results of basal and ligand-dependent nuclear import for the individual amino acids of the NLS indicate different roles for the two parts of the NLS and we hypothesized a difference in import mechanism for unliganded and ligand-bound AHR. We next intended to study the influence of importin specific inhibitors IPZ and IVM on the nucleo-cytoplasmic translocation of AHR.

**Figure 4:**
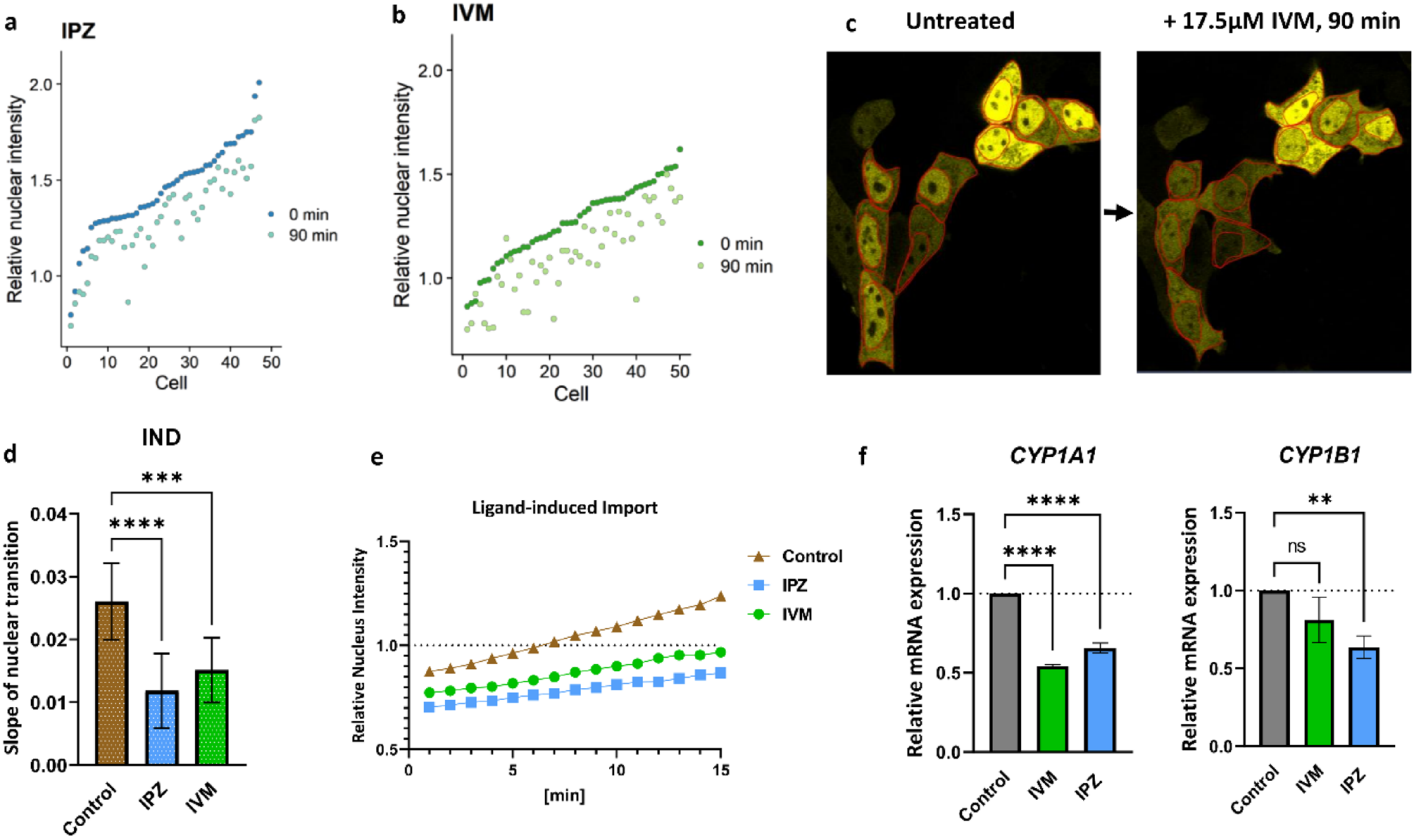
The inhibitors of importin (IMP) α/β mediated import importazole (IPZ) and ivermectin (IVM) reduce the nuclear accumulation of AHR. MCF-7^ΔAHR^ cells were transfected with EYFP-AHR^WT^ and treated with IPZ or IVM for 90 min at concentrations of 10 µM and 17.5 µM, respectively. Relative nuclear intensity of around 50 cells (sorted according to 0 min value) at the time of treatment (0 min) and after 90 min of treatment with IPZ (**a**) and IVM (**b**), respectively. Representative images of cells before and after incubation with 17.5 µM IVM for 90 min (**c**). Slopes of nuclear transition (**d**) and mean of time-lapse measurements (**e**) after incubation with 10 µM IPZ or 7.5 µM IVM for 22 h followed by co-treatment with 10 µM indirubin (IND) for 15 min. Data is expressed as mean + /− S.D. for 12 cells. Control implies to cells treated with ligand only. One-way ANOVA, Dunnett's post-test, ***p < 0.001, ****p < 0.0001. MCF-7^WT^ cells were treated with IPZ or IVM for 24 h at concentrations of 10 µM and 7.5 µM, respectively. Thereafter, cells were co-treated with 2.5 µM IND for 2 h. Relative *CYP1A1* and *CYP1B1* mRNA levels determined by qPCR (**f**). Values were standardized against HPRT and normalized to a sample treated with DMSO and ligand (mean + /− S.D.; one-way ANOVA, Dunnett’s post-test, **p < 0.01, ****p < 0.0001).

MCF-7^ΔAHR^ cells transiently transfected with EYFP-AHR^WT^ were incubated either with IPZ or IVM for 90 min. Image analysis elicited a significant reduction of the nuclear EYFP-AHR^WT^ staining. This was also accompanied with an increase of cytoplasmic EYFP-AHR^WT^ in studied cells. Therefore, the relative nuclear intensity diminished to 80% during this treatment (Fig. 4a–c and Supplementary Fig. S9).

Further analysis examined the influence of IPZ and IVM on the ligand-induced nuclear import of AHR. In response to IND stimulation, EYFP-AHR^WT^ live tracking exhibited remarkable deceleration of around 50% of nuclear import for both IPZ and IVM compared with the control (Fig. 4d,e). Taken together, these results indicate that basal and ligand-induced nuclear import are both affected by application of both import inhibitors.

To evaluate whether suppressing the AHR nuclear entry will influence AHR activity, we determined the functional output of the AHR after ligand stimulation either with or without preincubation with import inhibitors.

Compared to ligand stimulated control without import inhibitors, IVM treatment resulted in a notable decrease of *CYP1A1* mRNA levels and a slight decrease of *CYP1B1* mRNA expression (Fig. 4f)*.* On the other hand, IPZ resulted in a significant decrease in the expression of both *CYP1A1* and *CYP1B1* (Fig. 4f).

## Discussion

For import and export through the nuclear envelope, proteins larger than 40 kDa require specific signals in the form of amino acid sequences^42^. These signals permit recognition by nuclear transporters like the IMPα/β1 heterodimer for nuclear import or CRM1 for nuclear export. AHR possesses NLS and NES clusters, which unsurprisingly results in the ability of dynamic shuttling. Certainly, in the literature AHR is repeatedly defined as a cytoplasmic protein, while the nuclear entry requests ligand binding that induces a conformational change unmasking the NLS^43^^,^^44^^,^^45^. This theory ignores the nuclear retention of unliganded receptor detected in various in vitro systems confirming the existence of basal shuttling. Additionally, a nuclear trapped AHR shares a similar ability to bind ligands and induce *CYP1A1* expression in comparison to wild type AHR^29^. From our point of view, a mainly cytoplasmic localization could be attributed to high export efficiency or impeded import in individual cells. One popular theory links the basal nuclear accumulation of AHR with the presence of endogenous ligands in cell culture medium^46^. Nevertheless, a study of our lab showed that a part of ligand binding domain of AHR, namely the amino acid H291, is essential to bind endogenous ligands but is not mandatory for the nucleo-cytoplasmic shuttling^47^

Our first results confirmed that AHR subcellular localization of endogenous or overexpressed AHR varies between neighboring cells. From a technical aspect, cells in 2D cell culture are normally not synchronized and follow their own cell cycle progression. This offers the possibility that the noticed receptor staining might be related to various cell cycle stages. Especially since the cell cycle influences many physiological processes. In fact, in MCF-7 cells it has been shown that AHR physically interacts with proteins implicated in the cell cycle, like cyclin-dependent kinase 4 (CDK4)^48^ or the inhibitor of CDK2, p27^49^. More interesting, knocking out p27 resulted in increasing level of nuclear AHR^50^. In comparison to our results, it is interesting to note that the increase of detected cells in G1 phase in AHR positive cells might be related to AHR function. However, transfecting the MCF-7 cells with EYFP leads to a similar increase in the G1 phase, referring to a transfection-related effect on the cell cycle in general. Concisely, in our experiments typical distribution of AHR has been observed independent of cell cycle, expression level, or fusion with EYFP. Based on these data, we could make sure that we our system is suitable to study AHR localization.

In a previous study of our lab, we examined the DNA binding motif which has one overlapping amino acid with the 2nd NLS (EYFP-AHR^R40D^). This substitution impairs DNA binding and basal import, thereby changing the intracellular distribution^28^. Interestingly, this point mutation does not interfere with the ligand-induced import, which led us to hypothesize that two different import mechanisms for the AHR nuclear entry exist^28^.

Initially, Ikuta and co-workers identified the minimal NLS of AHR as _13_RKRR_16_ and _37_KRH_39_ by analyzing the intracellular distribution^12^. Our results expand the NLS sequence to include Arg17 along with Arg40 considering the localization of unliganded AHR. At the same time, His39 and Arg42 do not seem to influence the nuclear import in general. Technically, the methods used about 2 decades ago covered only short AHR variants involving the NLS and neighboring amino acids^12^^,^^30^. But this is not sufficient for full characterization of the AHR, since the C-terminus has an effect on its translocalization^31^. In particular, our experiments highlight a central role of the 1st NLS for the basal nuclear accumulation and the ligand-induced import of the receptor. Point mutations in the 2nd NLS lead to predominant cytoplasmic AHR staining, pointing to a diminished basal import, with only a slight reduction of the ligand-induced import.

Nuclear import can occur through IMPβ1 alone or with help of the adaptor protein IMPα^51^^,^^52^. According to Petrulis et al., murine AHR can be bound by IMPβ1 directly^18^. In combination with the apparently differing mechanisms for basal and ligand-induced import, we wanted to elucidate whether IMPα is mandatory for AHR translocation. IVM and IPZ are both specific inhibitors for IMP mediated import. IVM impedes binding between IMPα and NLS, thereby disrupting IMPα/β1 mediated transport^38^^,^^39^. IPZ disrupts IMPβ1 RanGTP binding, through which all IMPβ1 associated imports are inhibited^40^. Both inhibitors clearly reduce the basal nuclear accumulation of EYFP-AHR and diminish the ligand-induced import rate indicating similar mechanisms for both import processes.

**Figure 5:**
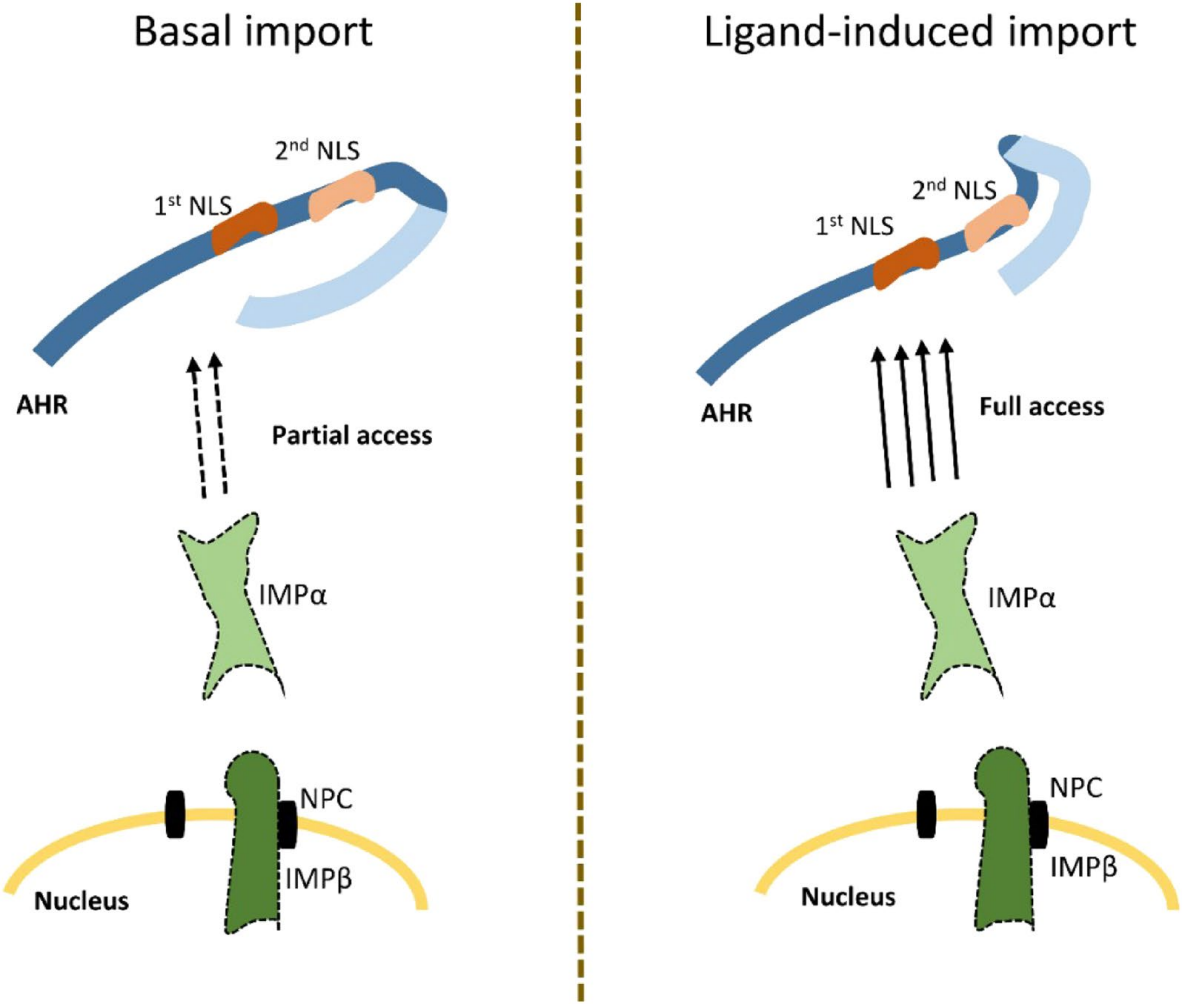
Nuclear import of AHR is mediated by importin (IMP) α/β1. The basal import occurs at a diminished rate because of a partial blocking of the 1st and 2nd nuclear localisation signal (NLS) through the c-terminal part of AHR or parts of the chaperon complex. Upon ligand binding, a conformational change grants full access for IMPα to the 1st NLS, thereby increasing import rate drastically. NPC, nuclear pore complex.

According to our results, we suggest the following hypothesis: In the unliganded state, blocking of both NLS parts is only partial, thereby allowing IMPα binding only at a diminished rate. The part of the AHR chaperone complex that is responsible for the shielding still needs to be determined. After ligand binding, the conformational change facilitates rapid IMPα recruitment without impediments. Interestingly, ligand-induced import is mainly implemented through the 1st NLS. The 2nd NLS is complementary but not essential. In conclusion, we show that ligand-independent nuclear import is mediated by the same molecular mechanism as the ligand-dependent import (Fig. 5).

## Methods

### Plasmids

The plasmid pEYFP-AHR-C1 has been used previously^28^ and encodes the human AHR starting with the amino acid alanine from position 11. GenScript (GenScript Biotech, Leiden, Netherlands) generated the modified variants of pEYFP-AHR that contain mutations at specific amino acids AHR^R13D^, AHR^K14D^, AHR^R15D^, AHR^R16D^, AHR^K17D^, AHR^1st.NLS^, AHR^K37D^, AHR^R38D^, AHR^H39D^, AHR^R40D^, AHR^R42D^ and AHR^2nd.NLS.^). AHR^WT^ and AHR^mut^ have been validated by sequencing (Eurofins, Germany).

### Cell culture

MCF-7 cell line was purchased from ATCC (Manassas, VA, USA). AHR knockout MCF-7 cells (MCF-7^∆AHR^) were generated in our lab using a CRISPR-Cas9 based approach^41^. Both cell lines were cultured in DMEM supplemented with 10% (v/v) FCS, 100 U/ml penicillin, 100 mg/ml streptomycin, and 2 mM L-glutamine. All cell culture media and supplements were bought from Pan-Biotech (Aidenbach, Germany). Cells were maintained in 5% CO_2_ at 37 °C and humidified atmosphere. Cells were routinely inspected for absence of mycoplasma contamination.

### Reagents

Β-naphthoflavone (BNF)**,** dimethyl sulfoxide (DMSO) and ivermectin (IVM) were acquired from Sigma-Aldrich (Sigma-Aldrich Chemie GmbH, Munich, Germany). Importazole (IPZ) was obtained from BIOZOL (Biozol Diagnostica Vertrieb GmbH, Eching, Germany). Indirubin (IND) was purchased from Enzo Life Sciences (Enzo Life Sciences GmbH, Lörrach, Germany). Leptomycin B (LMB) was deliviered from Santa Cruz Biotechnology (Santa Cruz Biotechnology Inc., TX, USA). DMSO was used as vehicle for BNF, IND, IPZ and IVM, while ethanol (Carl Roth, Germany) was used as a vehicle for LMB. The end-concentration does not exceed 0.1% when using DMSO or ethanol. All chemicals were ordered at the highest available purity.

### Transient transfection

MCF-7 or MCF-7^∆AHR^ cells were seeded either on 6-well plates (both from Techno Plastic Products AG, Trasadingen, Switzerland) or on glass bottom dishes (In VitroScientific, Sunyvale, CA, USA). After 24 h, cells on multiwell plates were transfected with lipofectamine 2000 (Invitrogen, Carlsbad, CA, USA) and an appropriate DNA amount as indicated by the manufacturer’s instructions. Meanwhile, cells on glass bottom dishes were transfected with Xfect (Takara Bio Europe SAS, Saint-Germain-en-Laye, France) and an appropriate DNA amount as stated by the manufacturer’s instructions. In both cases, transfection medium was refreshed after incubation for 4 h.

### Cell treatment with import inhibitors

To investigate the influence of import inhibitors on the basal localization of AHR, EYFP-AHR^WT^ transiently transfected MCF-7^ΔAHR^ cells were incubated with IPZ or IVM at concentration of 10 µM and 17.5 µM, respectively, for 90 min. To study the impact on ligand induced import, cells were incubated with 10 µM IPZ or 7.5 µM IVM after medium change after transfection for nearly 22 h and then co-treated with 10 µM IND for 15 min. To measure the effect of import inhibitors on the transcriptional activity of AHR, total RNA levels of MCF-7^WT^ cells were gathered after incubating the cells for 22 h with either IPZ or IVM at concentration of 10 µM and 7.5 µM, respectively, and afterwards stimulated with 2.5 µM IND for 2 h.

### Immunofluorescence staining

Cover glasses were coated with Poly-L-Lysin 0.01% solution (Sigma-Aldrich, Germany) according to the manufacturer’s instructions. Cells were grown on cover glasses with a cell density of 2.5 × 10^5^/ml for 48 h and then fixed in 3.7% formaldehyde (15 min), permeabilized with 0.2% Triton X-100 (Sigma-Aldrich, Germany) in PBS (10 min). Finally, cells were blocked with 5% fetal bovine serum (FBS) in PBS for 1 h at room temperature. After blocking, the coverslips were incubated with primary antibody for 90 min, washed and then incubated with the secondary antibody for 1 h. The antibodies were diluted in 1.5% FBS in PBS solution. Slides were mounted in Vectashield HardSet Mounting Medium with DAPI (VECTOR LABORATORIES, INC., Burlingame, CA, USA). The used primary antibody is anti-AHR (sc-5579, Santa Cruz Biotechnology, 1:25) and secondary antibody is goat anti rabbit, Alexa Fluor™ 594 (A-11012, 1:400), purchased from Invitrogen, Thermo Fisher Scientific, Germany.

### Cell cycle analysis by flow cytometry

Cells grown on glass bottom dishes were transfected using Xfect as described above. After 24 h, cells were detached from culture flasks by using trypsin (0.5%-EDTA 0.2%; Pan-Biotech (Aidenbach, Germany)) solution for 20 min. After cell fixation with ice cold 80% ethanol for 1 h, cells were washed with PBS and stained with propidium iodide (PI)/RNAse solution (Thermo Fisher Scientific, Germany) according to the manufacturer’s protocol. Stained cells were analyzed by fluorescence-activated cell-sorting (FACS) (BD FACSAria III). For data analysis, FlowJo (V.10.7.1, BD Biosciences) was used. For analysis, samples were analyzed to discriminate doublets (FSC-A versus FSC-W). Cell cycle analysis was performed using the PI-A intensity on a linear scale of either AHR-YFP-positive or -negative cells. To determine the intensity of G2-phase and verify cell cycle analysis, nocodazole (0.6 µg/ml; Sigma-Aldrich, Germany) synchronized cells were analyzed as positive control.

### Gene expression analysis

Total RNA was isolated form cells using RNeasy Midi kit and QIAshredder (both from QIAGEN GmbH, Hilden, Germany). Thereafter, purity and concentration of isolated RNA were determined using a plate reader device (Infinite M200 PRO, Tecan Trading AG, Männedorf, Switzerland). 1 µg or 500 ng of the extracted RNA was reversely transcribed into cDNA by using the high-capacity cDNA Reverse transcription kit (Applied Biosystems, Foster City, CA USA).

Transcript levels were determined with fast SYBR Green mix (Applied Biosystems, Foster City, CA, USA) and quantitative PCR (qPCR) device 7500 Fast Real-Time PCR instrument (Applied Biosystems, Foster City, CA, USA). Hypoxanthine–guanine phosphoribosyl transferase (*HPRT*) was used as reference gene.

The primer sequences are listed in Table 1.

**Table 1:** Primer sequences used.

Gene	Forward primer	Reverse primer
*CYP1A1*	5′-CCAAGAGTCCACCCTTCCCAGCT-3′	5′-GAGGCCAGAAGAAACTCCGTGGC-3′
*CYP1B1*	5′-TGGATTTGGAGAACGTACCG-3′	5′-CCACGACCTGATCCAATTCT-3’
*HPRT*	5′-GTTCTGTGGCCATCTGCTTAG-3′	5′-GCCCAAAGGGAACTGATAGTC-3′

### On-line confocal microscopy

As described in^28^, for cell monitoring the confocal microscope LSM 700 (Carl Zeiss Jena GmbH, Jena, Germany) was used. During the measurement of living cells, suitable cell culture conditions like 5% CO_2_ and 37 °C were maintained. The relative nuclear intensity refers to the mean fluorescence intensity of the nucleus divided by the mean fluorescence intensity of the whole cell. This was determined by outlining the nucleus and whole cells as region of interest (ROI) by using ZEN 2012 blue edition (Carl Zeiss Jena GmbH). The slope of nuclear transition was measured after plotting the relative nucleus intensity against time of the treatment. Representative examples of the analysis of AHR localization, time-lapse measurements and relative nuclear intensity are in (supplementary Figs. S1, S4 and S7).

### Statistics

Data analysis and graphing were performed with GraphPad Prism (Graph Pad, La Jolla, CA, USA) and R-Studio (R-Tools Technology Inc, Canada). Statistical analysis was done using paired Dunnett’s multiple comparisons test, one-way ANOVA, *p < 0.01, **p < 0.01, ***p < 0.001, ****p < 0.0001.

### Supplementary Information

Supplementary Figures.

## Data Availability

The datasets used and/or analysed during the current study available from the corresponding author on reasonable request.
