# Supplementary material for: The nuclear entry of the aryl hydrocarbon receptor (AHR) relies on the first nuclear localization signal and can be negatively regulated through IMPα/β specific inhibitors
Source: Sci Rep. 2023 Nov 11;13:19668. doi: 10.1038/s41598-023-47066-z (PMC10640566; doi:10.1038/s41598-023-47066-z)
Supplement: Supplementary file 1 — Supplementary Figures. [file 41598_2023_47066_MOESM1_ESM.pdf]

Supplementary information to

The nuclear entry of the aryl hydrocarbon receptor (AHR) relies on the first nuclear localization signal and can be negatively regulated through IMPα/β specific inhibitors

Rashad Haidar[1-2]*, Reneh Shabo[1], Marie Moeser[1], Andreas Luch[1-2] and Josephine Kugler[1]

[1] German Federal Institute for Risk Assessment (BfR), Department of Chemical and Product Safety, Berlin, Germany

[2] Institute of Pharmacy, Department of Biology, Chemistry and Pharmacy, Freie Universität Berlin, Berlin, Germany

*** Correspondence should be addressed to Rashad.Haidar@bfr.bund.de**

Supplementary Figures

a

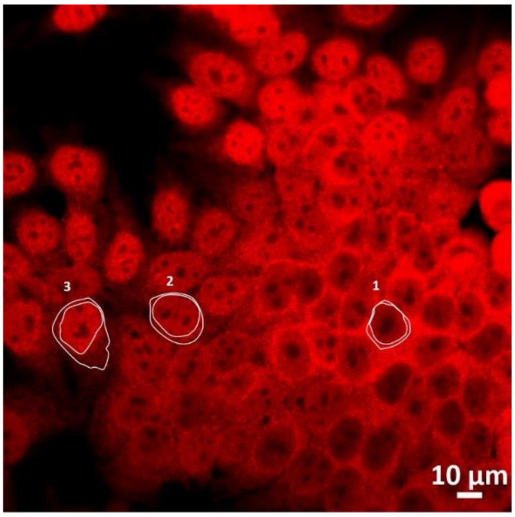

Name	Area	Fluorescence Intensity
	nm²	
Nucleus (1)	1,79735E+08	28,40085
Whole Cell (1)	2,30214E+08	32,49268
Nucleus (2)	2,43866E+08	30,58789
Whole Cell (2)	3,41581E+08	29,94433
Nucleus (3)	2,44534E+08	34,81347
Whole Cell (3)	4,52608E+08	29,06058

Cell	Nucleus	Whole Cell	Relative nucleus intensity
1	28,40085	32,49268	0,874069
2	30,58789	29,94433	1,021492
3	34,81347	29,06058	1,197962

b

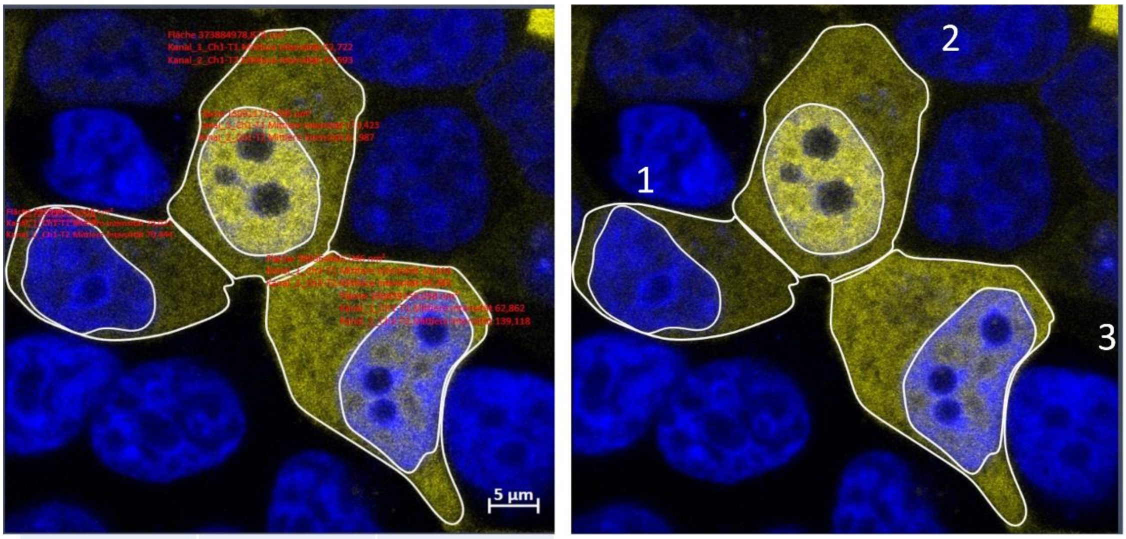

Name	Area	EYFP Intensity
	nm²	
Nucleus (1)	125434680	17,9864225
Whole Cell (1)	252944319	29,0213524
Nucleus (2)	150911716	110,42257
Whole Cell (2)	373884979	82,7222964
Nucleus (3)	389364605	70,4138841
Whole Cell (3)	166839155	62,8621562

Cell	Nucleus	Whole Cell	Relative nucleus intensity
1	17,9864225	29,0213524	0,61976514
2	110,42257	82,7222964	1,33485862
3	62,8621562	70,4138841	0,89275229

Supplementary Figure S1: Representative example of the assessment of relative nucleus intensity. The nucleus and the whole cells are marked as region of interest (ROI) by using ZEN 2012 blue edition. The relative nuclear intensity is

determined by the mean fluorescence intensity of the nucleus divided by the mean fluorescence intensity of the whole cell (a) Immunofluorescence images of AHR in MCF 7^{WT}. (b) EYFP-AHR^{WT} in transiently transfected in MCF $7^{\Delta AHR}$ cells.

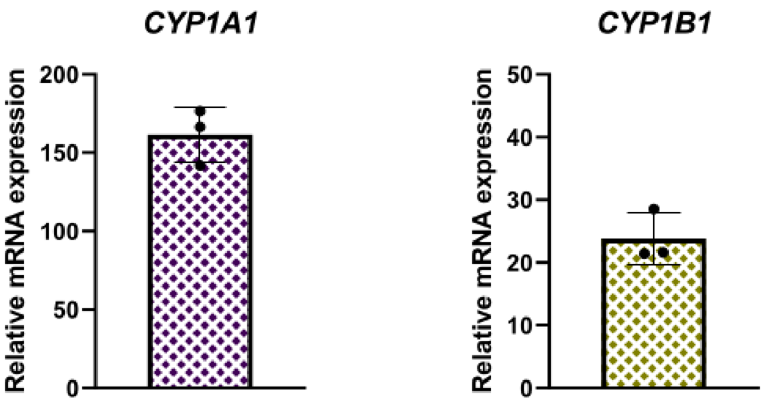

Supplementary Figure S2: Relative *CYP1A1* and *CYP1B1* in EYFP-AHR^{WT} transfected MCF-7 $^{\Delta AHR}$ cells. Cells were treated with 2.5 µM IND or 0.1% DMSO for 2 h. Relative *CYP1A1* and *CYP1B1* mRNA levels determined by qPCR. Values were standardized against HPRT and normalized to sample treated with DMSO only.

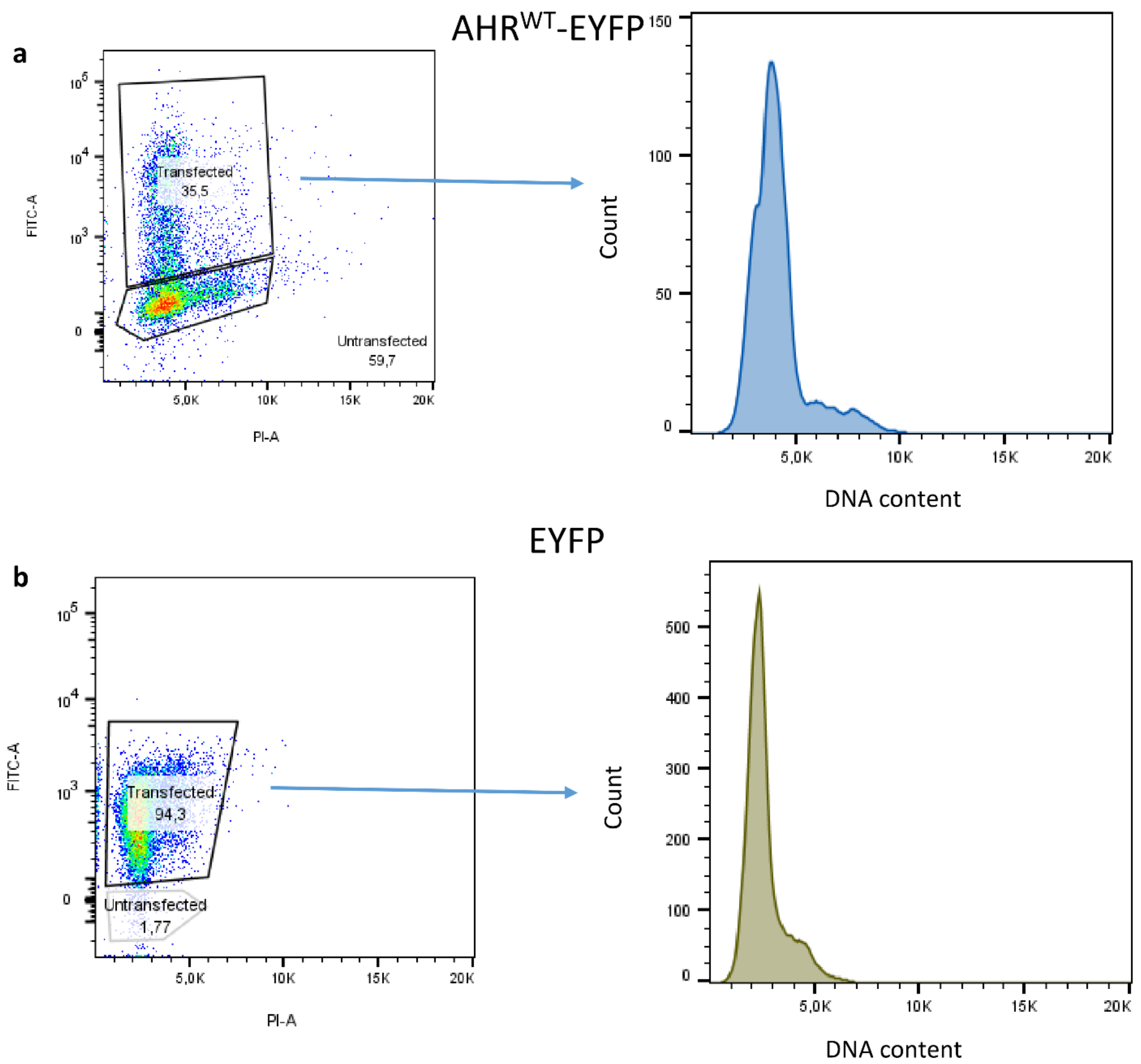

Supplementary Figure S3: Cell cycle analysis of (a) EYFP-AHR transfected and (b) EYFP-transfected cells. Total number of analyzed cells: 3573(a), 9715(b).

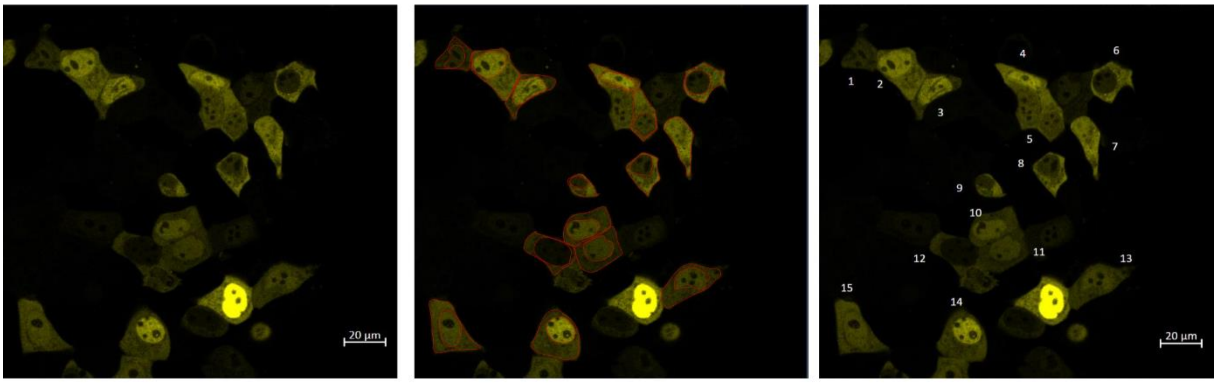

Cell	Nucleus	Whole Cell	Relative nucleus intensity	Localization
**1**	32,60259089	31,00435768	1,051548664	Equal distributed
**2**	94,34107402	79,82384993	1,181865747	Nuclear
**3**	92,80375147	72,89917012	1,273042633	Nuclear
**4**	112,2188771	95,00307161	1,181213146	Nuclear
**5**	48,92213533	60,3714155	0,81035263	Cytoplasmic
**6**	31,65004156	52,62605932	0,601413862	Cytoplasmic
**7**	88,76074435	86,60516028	1,024889788	Equal distributed
**8**	36,46220615	55,885867	0,652440556	Cytoplasmic
**9**	34,46927374	50,1973051	0,686675782	Cytoplasmic
**10**	58,87929677	48,69960793	1,209030201	Nuclear
**11**	47,60108884	38,58784858	1,233577164	Nuclear
**12**	13,51727057	20,60690953	0,655958165	Cytoplasmic
**13**	50,89350269	44,7212931	1,138015007	Nuclear
**14**	104,8081382	67,04128846	1,56333717	Nuclear
**15**	55,51982571	52,75499381	1,052408913	Equal distributed

Supplementary Figure S4: Representative example of determining the localization of EYFP-AHR^{WT}. MCF7$^{\Delta AHR}$ cells transfected with EYFP-AHR^{WT}. In the basal state, fifteen cells were selected and the relative intensity of the nucleus of each cell was measured. The localization of AHR was assigned according to our classification in Fig2h.

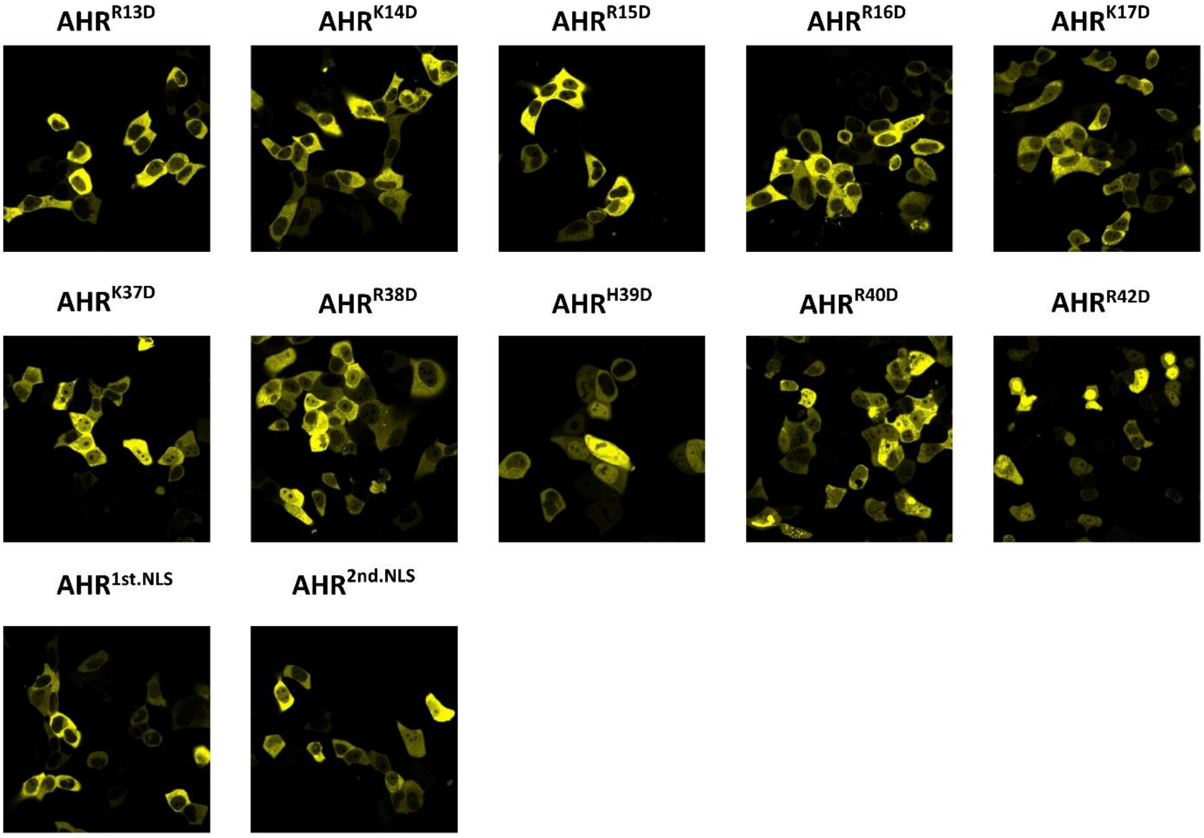

Supplementary Figure S5: Representative images of MCF7$^{\Delta AHR}$ cells transfected with EYFP-AHR^{mut} in the basal state.

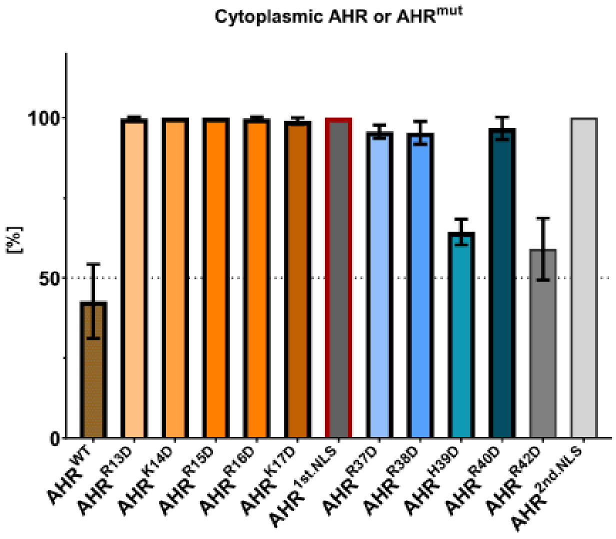

Supplementary Figure S6: The percentage of cells with cytoplasmic AHR^{WT} or AHR^{mut} for hundred randomly chosen cells according to our classification in Fig S2 and in Fig2h.

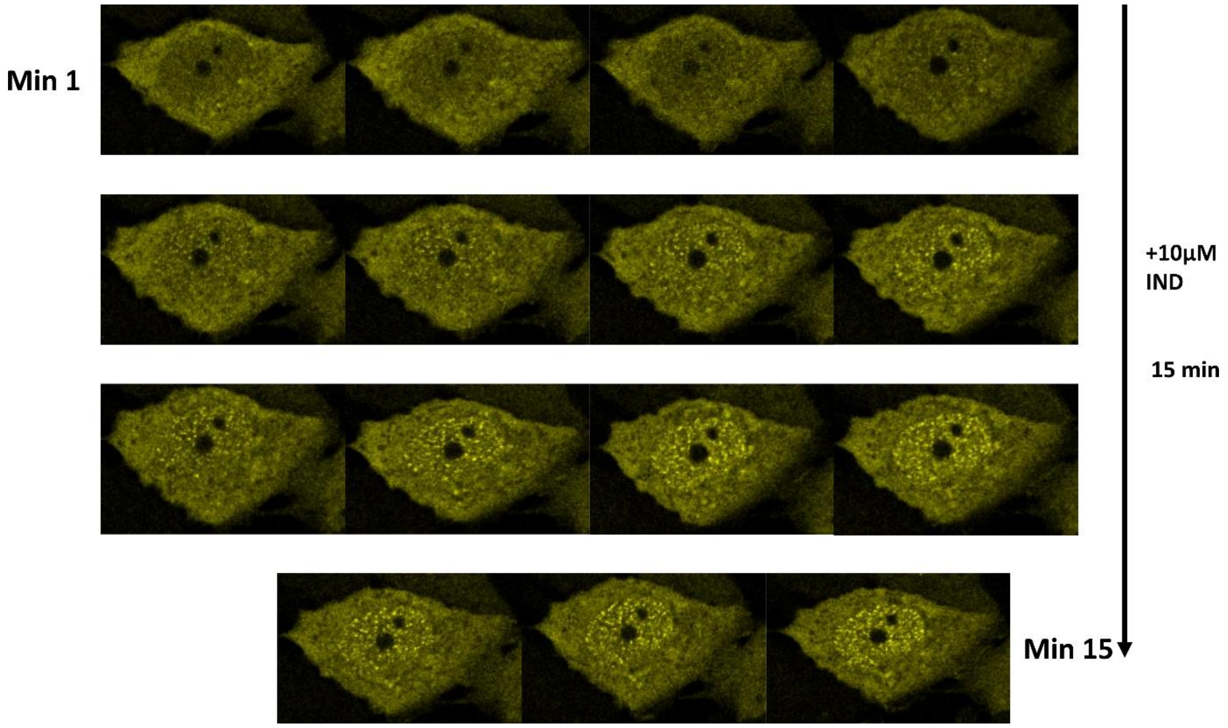

Time	Nucleus	Whole Cell	Relative Nucleus Intensity
1	9,21553719	10,4335161	0,88326285
2	9,01619835	10,1706648	0,88649056
3	8,28793388	9,15421522	0,90536804
4	10,7345455	10,9389993	0,98130964
5	10,1682645	10,6222298	0,9572627
6	10,7061157	10,8275074	0,98878858
7	11,8244628	11,2050491	1,05527987
8	12,6136489	11,3827964	1,1081327
9	12,2879339	10,8430432	1,13325509
10	12,7309091	11,2062828	1,13605102
11	13,911405	11,6710075	1,19196264
12	14,0634711	11,8881654	1,18298077
13	13,5110744	11,1554718	1,21116118
14	14,0502479	11,3701165	1,23571715
15	15,2241322	11,5685858	1,31598905

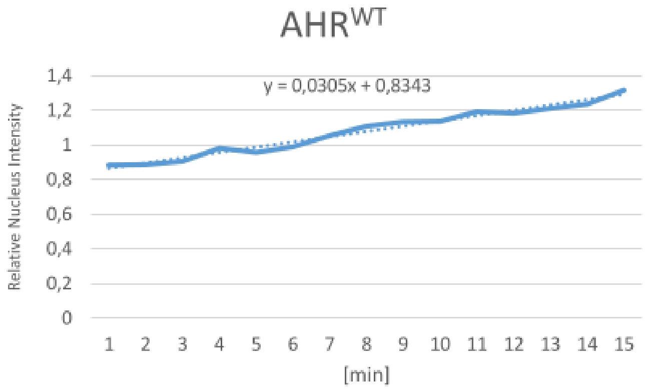

Slope of nuclear transition: 0,0305

Supplementary Figure S7: Calculation of the slope of nuclear transition after treatment. The representative example demonstrates the increase of relative nucleus intensity after cells treatment with 10 µM indirubin (IND) for 15 minutes. Fifteen snapshots of the cell are taken in ratio of one image per minute after treatment.

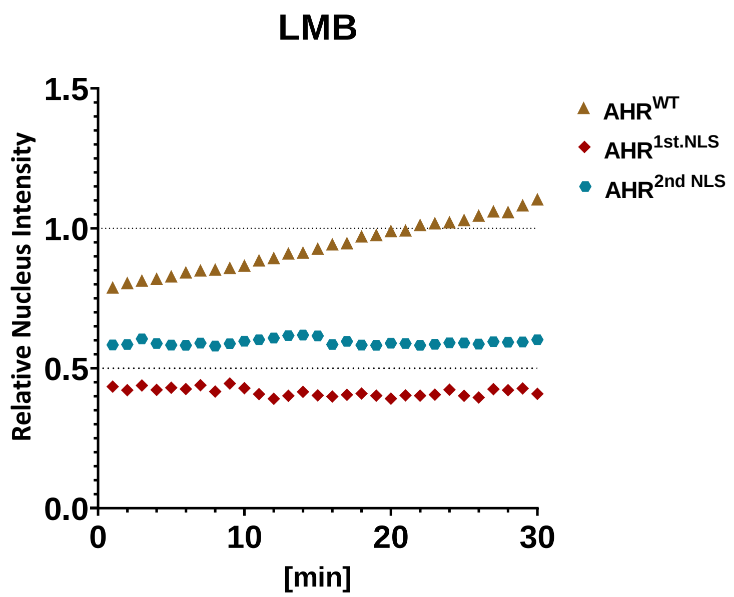

Supplementary Figure S8: Mean of time-lapse measurements after incubating cells transfected with AHR^{WT} or AHR^{mut} with 200 nM LMB for 30 min.

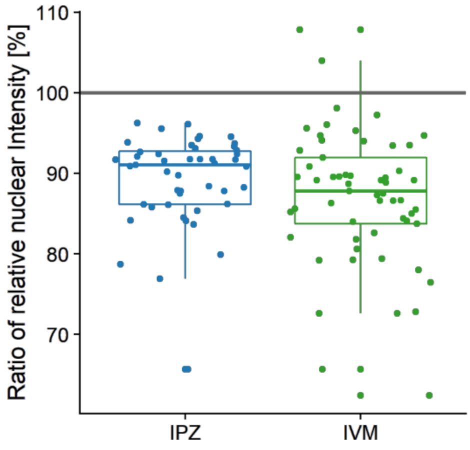

Supplementary Figure S9: Changes in the relative nuclear intensity after import inhibitors treatment. MCF7$^{\Delta AHR}$ cells were transfected with EYFP-AHR^{WT} and treated with importazole (IPZ) or ivermectin (IVM) for 90 min at concentrations of 10 µM and 17.5 µM, respectively. Changes in relative nuclear intensity of around 50 cells after 90 min of treatment are represented.
